# Cardiac remodeling in patients with childhood-onset craniopharyngioma*—*results of HIT-Endo and KRANIOPHARYNGEOM 2000/2007

**DOI:** 10.1007/s00431-020-03915-x

**Published:** 2021-01-18

**Authors:** Panjarat Sowithayasakul, Leona Katharin Buschmann, Svenja Boekhoff, Hermann L. Müller

**Affiliations:** 1 Department of Pediatrics and Pediatric Hematology/Oncology, University Children’s Hospital, Carl von Ossietzky University Oldenburg, Klinikum Oldenburg AöR, 26133 Oldenburg, Germany; 2 Department of Pediatrics, Faculty of Medicine, Srinakharinwirot University, Bangkok, 26120 Thailand

**Keywords:** Craniopharyngioma, Obesity, Hypothalamus, Pituitary, Cardiology, Echocardiography

## Abstract

Hypothalamic obesity caused by childhood-onset craniopharyngioma results in long-term cardiovascular morbidity. Knowledge about clinical markers and risk factors for cardiovascular morbidity is scarce. A cross-sectional study on transthoracic echocardiographic parameters was performed to determine the associations with clinical and anthropometric parameters in 36 craniopharyngioma patients. BMI correlated with the thickness of interventricular septum in diastole (IVSd) (*r* = 0.604, *p* < 0.001) and left ventricular posterior wall thickness in diastole (LVPWd) (*r* = 0.460, *p* = 0.011). In multivariate analyses on risk factors for cardiac remodeling, sex hormone replacement therapy, BMI, and male gender were positively correlated with increased left ventricular internal diameter in diastole (LVIDd), *R*^2^ = 0.596, *F* = 10.323, *p* < 0.001. BMI and insulin resistance were selected as significant independent determinants of IVSd, produced *R*^2^ = 0.655, *F* = 29.441, *p* < 0.001. Due to a wide range of disease duration, 17 pediatric and 19 adult patients were analyzed separately. In the adult subgroup (age at study ≥ 18 years), BMI correlated with IVSd (*r* = 0.707, *p* = 0.003), LVPWd (*r* = 0.592, *p* = 0.020), and LVIDd (*r* = 0.571, *p* = 0.026). In the pediatric subgroup (age at study < 18 years), no correlation between transthoracic echocardiography (TTE) parameters and BMI was observed. Only LVIDd correlated with disease duration (*r* = 0.645, *p* < 0.001). All cardiac functions were within the normal range, indicating no association with functional impairments.

*Conclusion*: Cardiac remodeling in patients with craniopharyngioma correlated with the degree of hypothalamic obesity, disease duration, sex hormone replacement therapy, male gender, and insulin resistance. As echocardiography has limited sensitivity in patients with obesity, further research on more sensitive techniques for cardiac diagnostics in craniopharyngioma patients is warranted.

**Table:** 

**What is Known:** *•Long-term prognosis in survivors of craniopharyngioma is impaired by obesity and cardiovascular disease.* *•Associations between echocardiographic findings and clinical and anthropometric parameters after craniopharyngioma are not yet analyzed.*	
**What is New:** *•In patients with childhood-onset craniopharyngioma, cardiac remodeling was associated with hypothalamic obesity, duration of disease, male gender sex hormone replacement, and insulin resistance.* *•Due to reduced echocardiographic sensitivity caused by obesity-related technical limitations, more sensitive cardiac diagnostics should be considered.*

**What is Known:**

*•Long-term prognosis in survivors of craniopharyngioma is impaired by obesity and cardiovascular disease.*

*•Associations between echocardiographic findings and clinical and anthropometric parameters after craniopharyngioma are not yet analyzed.*

**What is New:**

*•In patients with childhood-onset craniopharyngioma, cardiac remodeling was associated with hypothalamic obesity, duration of disease, male gender sex hormone replacement, and insulin resistance.*

*•Due to reduced echocardiographic sensitivity caused by obesity-related technical limitations, more sensitive cardiac diagnostics should be considered.*

## Introduction

Craniopharyngiomas (CPs) are rare tumors with an incidence of 0.5–2 cases per million people per year [1, 2]. The most common locations of CP are the sellar and suprasellar regions [3]. CPs are histologically benign and classified as WHO grade I tumors [4]. Two primary subtypes have been recognized from histological studies: the adamantinomatous and the papillary type [5]. Disease- and/or treatment-related damage to hypothalamic structures has been associated with higher mortality and morbidity. The most appropriate treatment option for CP patients with favorable tumor location is tumor resection with preservation of hypothalamic and optical functionality. Limited resection followed by local irradiation is recommended when CP tumors involve hypothalamus or optic structures [3, 6, 7]. The long-term morbidity after CP is characterized by the involvement of (neuro) endocrine structures, visual disturbances, hypothalamic damage, neurobehavioral and cognitive sequelae [1, 8]. Previous reports have shown that up to 50% of CP survivors suffer from obesity due to hypothalamic involvement (HI) of the tumor at moment of diagnosis or hypothalamic damage resulting from surgical interventions [8, 9]. Patients with initial HI had a higher standardized body mass index (BMI SDS) already at the time of diagnosis and during annual follow-up when compared with patients presenting at diagnosis with a CP not involving hypothalamic structures [10]. The hypothalamus plays an important role in the regulation of energy homeostasis and circadian rhythms [11]. Accordingly, hypothalamic nuclei injury results in morbid obesity frequently accompanied by fatigue, decreased physical activity, and disturbances of satiety and hunger [12]. Moreover, hypothalamic obesity increases the risk of metabolic syndrome, cardiovascular disease including acute cardiac death, multisystemic morbidity, and multisystemic mortality [7]. A 22% rate of long-term cardiovascular complications was reported in patients with CP, associated with an almost 3-fold increased risk of mortality [13, 14].

Cardiac remodeling is a term used to describe physiologic and pathologic changes that may affect size, mass, and function of the heart due to several etiologies. Myocardial remodeling caused by obesity leads to subsequent development of heart failure [15].

In our study, we focused on pathological cardiac remodeling in CP patients [16, 17]. Direct correlations between the BMI and cardiac remodeling have been reported, observing an increased cardiac septum and left ventricular posterior wall thickness in patients with obesity [18, 19]. The aim of our research was to study structural cardiac abnormalities in patients with CP and hypothalamic obesity and its association with clinical and anthropometric parameters.

## Patients and methods

Cardiac status was analyzed by transthoracic echocardiography (TTE) in 36 out of 675 patients with childhood-onset, adamantinomatous CP recruited in HIT-Endo and KRANIOPHARYNGEOM 2000/2007 to determine associations with clinical and anthropometric parameters. As TTE is not part of the suggested follow-up program in the multicenter trials KRANIOPHARYNGEOM 2000/2007, all patients with CP who were treated between 2015 and 2016 at the outpatient Department of the University Children’s Hospital, Klinikum Oldenburg AöR, Oldenburg, Germany, were included in our cross-sectional study. Body weight and height were measured by using a weighing scale and a Harpenden stadiometer. BMI and BMI SDS values were calculated and expressed according to the references of Rolland-Cachera et al. [20]. The CP patients were classified as severely obese, obese, and normal-weight if their BMIs were > 8 SDS, + 2 to + 8 SDS, and − 2 to + 2 SDS, respectively. TTE was performed by using a Vivid E9 (GE Healthcare, General Electric company; Buckinghamshire, UK) with a 3, 6, and 12-MHz transducer. Two-dimensional M-mode measurements of the left ventricular internal diameter in diastole (LVIDd), interventricular septal thickness in diastole (IVSd), left ventricular posterior wall in diastole (LVPWd), and tricuspid annular plane systolic excursion (TAPSE) were performed by TTE. Additional parameters of left ventricular function (ejection fraction (EF) and fractional shortening (FS)) were calculated by using the following equations:

$$ {\displaystyle \begin{array}{l} FS=\frac{EDD- ESD}{EDD}\ \left( EDD= end\ diastolic\ dimension; ESD= end\ systolic\ dimension\right)\\ {} EF=\frac{SV}{EDV}\ \left( SV= stroke\ volume; EDV= end\ diastolic\ volume\right)\end{array}} $$

The result of the equations for FS and EF is given in percent. The normal ranges and severity cutoff values of TTE parameters are depicted in Table 2 [21]. The normal ranges and severity cutoff of TTE parameters were classified by gender in adult patients. All TTE parameters in pediatric patients as normalized by body surface area [22] were within the normal range, with the exception of one severe obese pediatric patient who was found to have abnormally increased LVPW thickness (data not shown).

Neuroradiological assessment of CP tumor location, degree of surgical resection, preoperative HI, and surgical hypothalamic lesions (HL) was performed on pre- and postoperative axial, coronal, and sagittal magnetic resonance imaging (MRI) as previously described [23, 24]. The histological diagnosis of adamantinomatous CP was confirmed by neuropathological reference assessment in all cases.

Statistical analyses were performed using IBM SPSS statistic program version 24.0. The differences between groups were assessed by Student’s *t* tests as well as the ANOVA (in the case where several categories were present). Pearson’s correlation coefficient was calculated to determine the relationship between two variables. Stepwise multivariate linear regression analysis was also performed to determine the independent predictors of TTE parameters: LVIDd and IVSd. Independent variables in the multivariate model included BMI, gender, sex steroid replacement, and insulin resistance status. The significance level is set to 0.05, that is, *p* values ≤ 0.05 were considered statistically noticeable. No adjustment for multiple testing was applied. Therefore, inferential statistics are intended to be exploratory (hypotheses generating), not confirmatory, and are interpreted accordingly.

## Results

A total of 36 (21 female/15 male) of 675 patients with CP recruited in HIT-Endo and KRANIOPHARYNGEOM 2000/2007 were included in our study. Our study cohort was comparable with the total group of patients registered in the abovementioned trials (Table 1). The median age at diagnosis was 8.8 years, ranging from 3.0 to 15.1 years. Six patients were prepubertal, 30 patients were pubertal or postpubertal at the time of the study. Median BMI SDS at the time of diagnosis was + 1.7 SDS, ranging from − 3.8 to + 9.9 SDS. Twenty-five patients (69%) presented with HI [23, 24] at the time of CP diagnosis. In 11 patients (31%), gross-total resection was achieved, whereas 20 patients (56%) underwent partial resection. Surgical HLs [23, 24] were referenced—confirmed by a neuroradiologist and blinded for clinical data in 15 patients (42%). Postoperative irradiation was performed in 10 patients (28%). Pituitary deficiencies were observed in 31 patients (86%) after surgery. Nineteen patients (53%) had endocrine deficiencies of more than four hypothalamic-pituitary axes. The median follow-up interval between CP diagnosis and the time of study was 8.8 years, ranging from 0.3 to 25.9 years (Table 1).

**Table 1:** Patient characteristics of 36 patients with childhood-onset, adamantinomatous craniopharyngioma (CP) (study cohort) and 675 patients with CP recruited in HIT-Endo and KRANIOPHARYNGEOM 2000/2007

	Total cohort (KRANIO 2000/2007, HIT-Endo)	Study cohort	Pediatric study cohort (age < 18 years)	Adult study cohort (age ≥ 18 years)
Patients, *n*	675	36	17	19
Gender, *n* (female/male)	333/342	21/15	9/8	12/7
Median age at CP diagnosis, years (range)	9.0 (0.1–27.0)	8.8 (3.0–15.1)	6.8 (3.0–16.0)	10.5 (5.3–15.1)
Median age at study, years (range)		18 (5.5–41.0)	12.3 (5.5–17.8)	24.7 (18.3–41.0)
Median follow-up interval, years (range)	5.7 (0.1–38.87)	8.8 (0.3–25.9)	3.7 (0.3–9.6)	12.3 (8.5–25.9)
Hypothalamic involvement (HI) [23], *n* (%)				
Anterior HI	63 (9)	11 (31)	6 (35)	5 (26)
Anterior and posterior HI	161 (24)	14 (39)	8 (47)	6 (31)
No HI	15 (2)	8 (22)	3 (18)	5 (26)
n.a.	436 (65)	3 (8)	0 (0)	3 (16)
Degree of surgical resection, *n* (%)				
Complete resection	187 (28)	11 (31)	3 (18)	8 (42)
Incomplete resection	384 (57)	20 (56)	11 (65)	9 (47)
No surgical intervention	7 (1)	1 (3)	0 (0)	1 (5)
n.a.	97 (14)	4 (11)	3 (18)	1 (5)
Hypothalamic lesion (HL) [23], *n* (%)				
Anterior HL	91 (14)	7 (19)	3 (18)	4 (21)
Anterior and posterior HL	83 (12)	8 (22)	4 (24)	4 (21)
No HL	67 (10)	18 (50)	10 (59)	8 (42)
n.a.	436 (65)	3 (8)	0 (0)	3 (16)
Number of substituted endocrine axes, *n* (%)				
None	17 (3)	4 (11)	4 (24)	0 (0)
One axis	25 (4)	2 (6)	2 (12)	0 (0)
2 axes	40 (6)	3 (8)	1 (6)	2 (11)
3 axes	110 (16)	7 (19)	4 (24)	3 (16)
4 axes	122 (18)	10 (28)	6 (35)	4 (21)
5 axes	54 (8)	9 (25)	0 (0)	9 (47)
n.a.	307 (45)	1 (3)	0 (0)	1 (5)
GH deficiency/GH substituted	255 (78)	24 (67)	8 (47)	16 (84)
TSH deficiency/thyroxin substituted	318 (89)	28 (78)	11 (65)	14 (74)
Irradiation, *n* (%)	242 (44)	10 (28)	2 (12)	8 (42)
BMI SDS [20] at study/last visit				
Normal weight (− 2 to + 2 BMI SDS)	250 (41)	13 (36)	7 (41)	6 (31)
Obesity (+ 2 to + 8 BMI SDS)	301 (49)	14 (39)	8 (47)	6 (31)
Severe obesity (> + 8 BMI SDS)	61 (10)	9 (25)	2 (12)	7 (37)

*BMI*, body mass index; *CP*, childhood-onset craniopharyngioma; *GH*, growth hormone; *HI*, hypothalamic involvement; *HL*, hypothalamic lesion; *n.a*, data not available; *SDS*, standard deviation score; *TSH*, thyroid-stimulating hormone

**Fig. 1:**
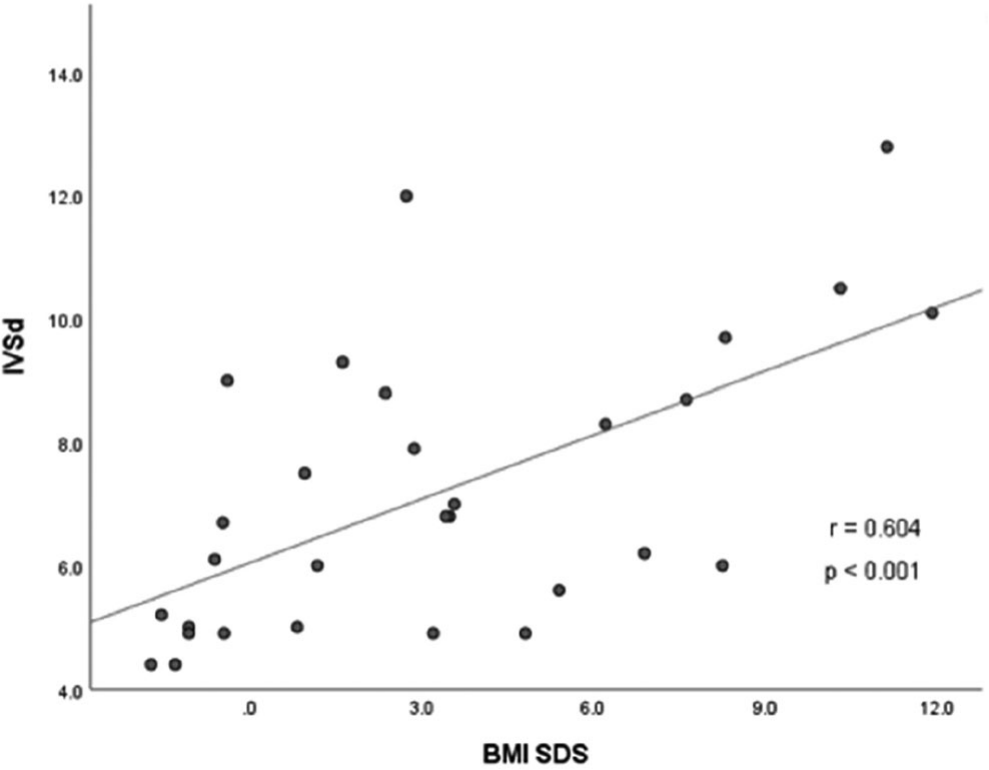
Correlation between the BMI SDS [20] and interventricular septal thickness in diastole (IVSd) in 30 patients with childhood-onset, adamantinomatous craniopharyngioma, and available data recruited in HIT-Endo and KRANIOPHARYNGEOM 2000/2007. BMI, body mass index; IVSd, interventricular septal thickness in diastole; SDS standard deviation score; r, Pearson correlation coefficient

**Fig. 2:**
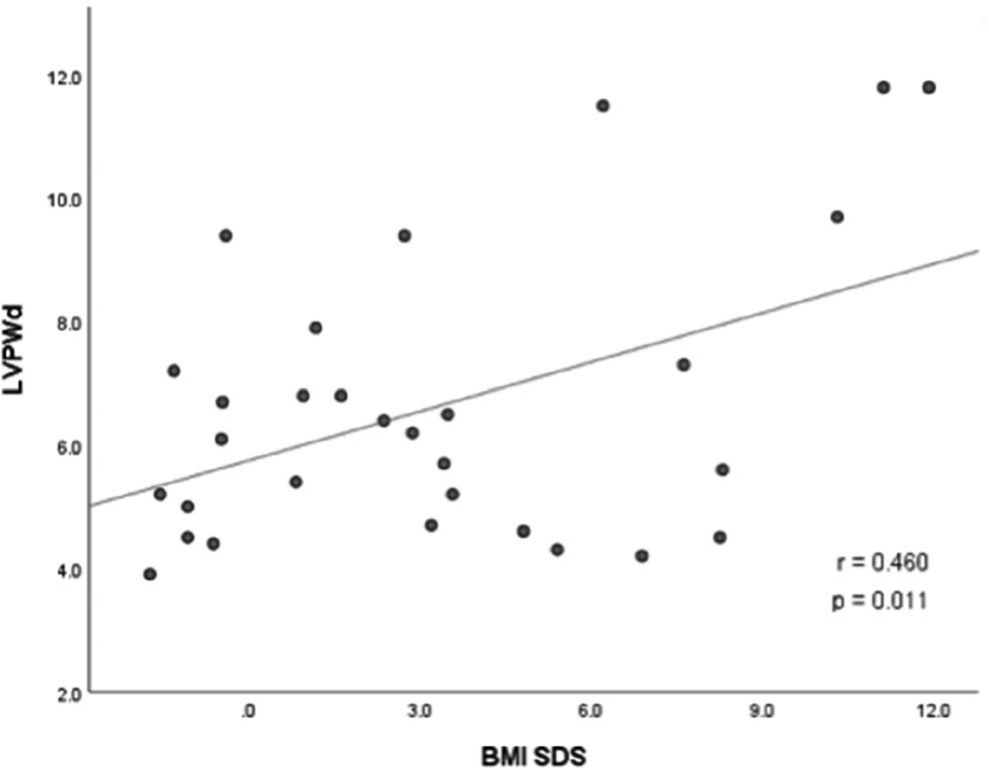
The correlation between BMI SDS [20] and left ventricular posterior wall thickness in diastole (LVPWd) in 30 patients with childhood-onset, adamantinomatous craniopharyngioma, and available data recruited in HIT-Endo and KRANIOPHARYNGEOM 2000/2007. BMI, body mass index; LVPWd, left ventricular posterior wall thickness in diastole; SDS, standard deviation score; r, Pearson correlation coefficient

**Fig. 3:**
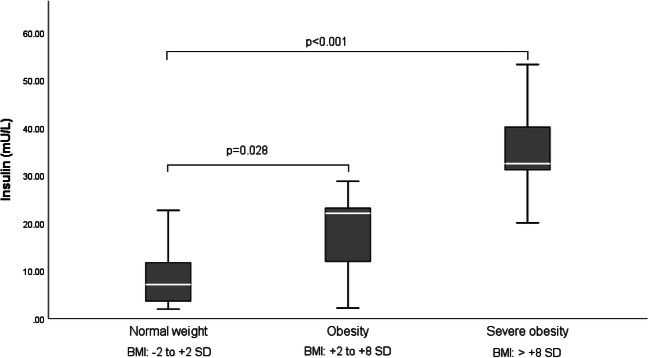
Insulin concentration (mU/L) in childhood-onset, adamantinomatous craniopharyngioma patients with normal weight (BMI − 2 to + 2 SDS), obesity (BMI + 2 to + 8 SDS), and severe obesity (BMI > +8 SDS). The horizontal line in the middle of the box depicts the median. The bottom edge marks the 25th percentile and the top edge marks the 75th percentile. Whiskers indicate the range of values that fall within 1.5 box-length

**Fig. 4:**
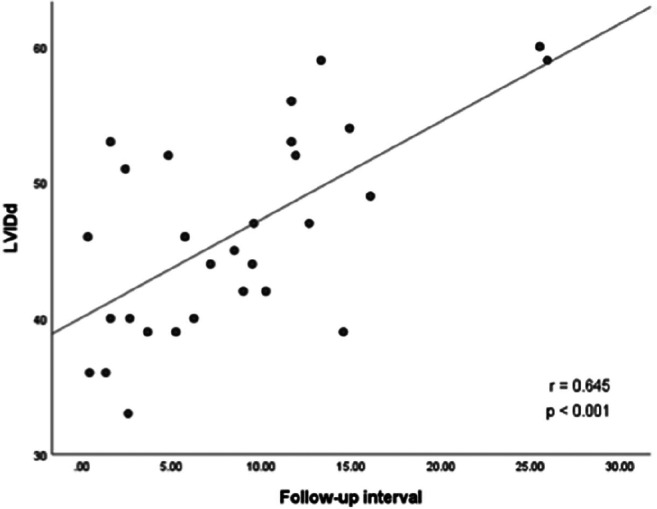
The correlation between follow-up interval and left ventricular internal diameter in diastole (LVIDd) in 29 patients with childhood-onset, adamantinomatous craniopharyngioma, and available data recruited in HIT-Endo and KRANIOPHARYNGEOM 2000/2007. LVIDd, left ventricular internal diameter in diastole; r, Pearson correlation coefficient

The association between TTE parameters and body composition parameters were analyzed. The strongest correlations were observed between IVSd and BMI SDS (*r* = 0.604, *p* < 0.001) (Fig. 1) and between LVPWd and BMI SDS (*r* = 0.460, *p* = 0.011) (Fig. 2). Differences in terms of TTE parameters were investigated in (post)pubertal patients with and without sex hormone replacement. CP patients with sex hormone replacement showed significant larger LVIDd compared to patients without sex hormone replacement (52.04 mm vs. 45.62 mm, *p* = 0.009). Insulin resistance (IR), which was classified by an age- and sex-specific homeostasis model assessment to quantify IR (HOMA-IR) [25, 26], was observed in 22 patients in this study. IR correlated with BMI and BMI SDS (*r* = 0.566, *p* = 0.001 and *r* = 0.664, *p* < 0.001, respectively). CP patients with severe obesity had significantly higher insulin levels (*p* < 0.001) compared to normal-weight and obese CP patients (Fig. 3). CP patients with IR had larger IVSd (mean of 8.71 vs. 5.50 mm, *p* < 0.001) and LVPWd (mean of 7.98 vs. 5.57 mm, *p* < 0.001) when compared to patients without IR. Moreover, IVSd correlated with the insulin level (*r* = 0.399, *p* = 0.019). Furthermore, potential associations between TTE parameters and other variables such as number of substituted endocrine axes, radiation therapy, hypertension, HL, HI, and follow-up interval were investigated. IVSd and LVPWd were correlated with HL (*r* = 404, *p* = 0.03 and *r* = 0.434, *p* = 0.019, respectively). No correlations between HI and TTE parameters were observed. Only LVIDd showed a correlation with the follow-up interval (*r* = 0.645, *p* < 0.001) (Fig. 4). No correlations were observed between hypertension and BMI (*r* = − 0.092, *p* = 0.593) or between hypertension and any TTE parameter (Table 2).

**Table 2:** Normal ranges and severity cutoff of TTE parameters [21]

TTE parameter	Male				Female			
	Normal	Slightly changed	Moderately changed	Severely changed	Normal	Slightly changed	Moderately changed	Severely changed
LVIDd, mm	42–58	59–63	64–68	> 68	38–52	53–56	57–61	> 61
IVSd, mm	6–10	11–13	14–16	> 16	6–9	10–12	13–15	> 15
LVPWd, mm	6–10	11–13	14–16	> 16	6–9	11–12	13–15	> 15
EF, %	52–72	41–51	30–40	< 30	54–74	41–53	30–40	< 30
FS, %	25–43	20–24	15–19	< 15	27–45	22–26	30–41	< 30
TAPSE, mm	24 ± 3.5	Abnormality threshold < 17			24 ± 3.5	Abnormality threshold < 17		

Interpretation of TTE parameters in pediatric patients was based on the cardiac structure dimensions normalized to the body surface area [22]; only one extreme obese pediatric CP patient was observed to present with abnormally increased LVPW thickness

*EF*, ejection fraction; *FS*, fractional shortening; *IVSd*, interventricular septal thickness in diastole; *LVIDd*, left ventricular internal diameter in diastole; *LVPWd*, left ventricular posterior wall thickness in diastole; *TAPSE*, tricuspid annular plane systolic excursion; *TTE*, transthoracic echocardiography

Multivariate regression analyses were performed to examine the relationship between TTE parameters and several potential predictors. In 30 (post)pubertal patients, 17 patients received sex steroid replacement therapy due to hypogonadism. Interestingly, sex steroid replacement therapy, BMI, and gender were associated with increased LVIDd. The multiple regression model with all three predictors produced *R*^2^ = 0.596, *F* = 10.323, and *p* < 0.001, respectively (Table 3), indicating that CP patients with sex steroid replacement, higher BMI, and male gender were expected to have larger LVIDd. Multivariate regression analysis was performed in 22 insulin-resistant patients. In all subjects, BMI and IR were selected as significant independent determinants of IVSd, which produced *R*^2^ = 0.655, *F* = 29.441, *p* < 0.001, indicating that CP patients with higher BMI and IR were expected to have larger IVSd.

**Table 3:** Multiple regression model showing correlation between LVIDd and patients with sex steroid replacement therapy, body mass index (BMI) SDS [20], and male gender

Variable	*B*	SE *B*	*β*	*t*	*p* value	95% CI	
						Upper	Lower
Patients with sex steroid replacement therapy (reference group: Patients without sex steroid replacement therapy)	4.599	1.863	0.366	2.469	0.022	0.725	8.472
BMI SDS [20]	0.705	0.230	0.455	3.068	0.006	0.227	1.183
Male (reference group: female)	4.785	1.744	0.381	2.744	0.012	1.158	8.412
Constant	42.006	1.574		26.683	0.000	38.732	45.280

*R*^2^ = 0.596, SEE = 4.35166, *F* = 10.323, Sig of *F* = 0.000

*B*, unstandardized beta; *β*, standardized beta; *BMI*, body mass index; *CI*, confidence interval; *LVIDd*, left ventricular internal diameter in diastole; *SDS*, standard deviation score; *SE B*, standard error for the unstandardized beta; *t*, *t* test statistic

At the time of study, 13 CP patients presented with normal weight (BMI: − 2.0 to + 2.0 SDS), while 14 patients showed obesity (BMI: + 2.0 to + 8.0 SDS), and nine patients had severe obesity (BMI: > + 8.0 SDS). Patients with obesity and severe obesity presented with significantly larger IVSd (*p* = 0.015) when compared with normal-weight CP patients (Table 4).

**Table 4:** Transthoracic echocardiographic features in normal-weight, obesity, and severe obesity in childhood-onset, adamantinomatous craniopharyngioma recruited in HIT-Endo and KRANIOPHARYNGEOM 2000/2007. Depicted are means and ranges

TTE parameter		BMI SDS, *n*		*p* value
	Normal weight, 13 (BMI − 2 to + 2 SDS)	Obesity, 14 (BMI + 2 to + 8 SDS)	Severe obesity, 9 (BMI > + 8 SDS)	
IVSd (mm), % abnormal value	4.6 (4.4–9.3), 8%	7.1 (4.9–12.0)^2md^, 8%	9.7 (6–12.8)^4md^, 44%	0.012, 0.015^#^
LVIDd (mm), % abnormal value	48.6 (33.4–58.6), 8%	53.1 (35.6–59.5)^2md^, 14%	46.2 (36.0–59.1)^3md^, 44%	0.161, 0.057^#^
LVPWd (mm), % abnormal value	5.3 (3.9–9.4), 0%	5.8 (4.2–11.5)^2md^, 8%	5.05 (4.5–11.8)^4md^, 33%	0.323, 0.586^#^
TAPSE, % abnormal value	22.5 (15.8–28.8)^5md^8%	25.6 (17.0–28.3)^8md^, 0%	25.8 (22.2–34.3)^3md^, 0%	0.356, 0.189^#^
EF (%), % abnormal value	68.0 (59–71), 0%	66.5 (62–74)^2md^, 0%	67.5 (64–74)^4md^, 0%	0.064, 0.020^#^
FS (%) % abnormal value	38.0 (30–40), 0%	36.5 (33–41)^2md^, 0%	37.0 (33–43)^3md^, 0%	0.148, 0.056^#^

*md*, missing data; *BMI*, body mass index; *EF*, ejection fraction; *FS*, fractional shortening; *IVSd*, interventricular septal thickness in diastole; *LVIDd*, left ventricular internal diameter in diastole; *LVPWd*, left ventricular diastolic posterior wall thickness in diastole; *TAPSE*, tricuspid annular plane systolic excursion; *SD*, standard deviation; *TTE*, transthoracic echocardiography

^#^*p* value of difference between the normal weight (BMI ≤ + 2 SDS) and the obese (BMI SDS > + 2 SDS) group

EF and FS were both within the normal range in all patients, without any association to BMI SDS. Of the 36 patients, TAPSE values were only measurable in 20 CP patients (56%), due to limited visualization of the tricuspid annulus in the obese CP patients. TAPSE showed no significant correlation with BMI SDS. TTE parameters could not be completely evaluated in 11 patients with obesity due to limited and impaired sound conditions by obesity.

Due to the large heterogeneity of our patient group with regard to follow-up intervals (ranging from 0.3 to 25.9 years), we analyzed TTE parameters for the subgroup of pediatric patients with an age < 18 years and adult patients with an age ≥ 18 years at the time of TTE.

In the adult subgroup, 19 patients with CP were diagnosed between 1990 and 2007, at a median age of 10.5 years. HI was diagnosed in 11 patients (58%) and HL in 8 patients (42%). Complete resection was achieved in 8 patients (42%), and postoperative irradiation was performed in 8 patients (42%). At the time of study, 19 adult patients presented with a median age of 24.7 years (range: 18.3–41.0 years), and a median follow-up interval between CP diagnosis and study of 12.3 years (range: 8.5–25.9 years). Six adult CP patients (32%) presented with normal weight, while 6 patients (32%) were obese, and 7 patients showed (36%) severe obesity (Table 1). In the adult subgroup, positive correlations with BMI SDS were observed for IVSd (*r* = 0.707, *p* = 0.003), LVIDd (*r* = 0.571, *p* = 0.026), and LVPWd (*r* = 0.592, *p* = 0.020). In adult patients with severe obesity, abnormally increased IVSd, LVIDd, and LVPWd were reported in 44%, 44%, and 33%, respectively. In addition, IVSd in the adult subgroup was significantly thicker than in the pediatric subgroup (8.18 mm vs. 6.18 mm, *p* = 0.016).

Our second subgroup consisted of 17 patients in the pediatric age group at the time of study, who were diagnosed between 2005 and 2015 at a median age of 6.8 years (range: 3.0–16.0 years). Fourteen patients (82%) had HI at initial CP diagnosis, and seven patients (41%) were observed with surgical HL. Complete resections were performed in 3 patients (18%), while incomplete resections were achieved in 11 patients (65%). Only two patients (12%) underwent postoperative irradiation. At the time of study, the 17 pediatric patients presented with a median age of 12.3 years, ranging from 5.5 to 17.8 years and a median follow-up interval of 3.7 years (range: 0.3–9.6 years) (Table 1). In the pediatric subgroup, no correlations were observed between TTE parameters and BMI SDS. All TTE parameters in pediatric patients as classified by body surface area [22] were normal, with the exception of one extreme obese pediatric patient who was found to have abnormally increased LVPW thickness.

## Discussion

The twenty-year outcome analyses in patients with CP recruited in the German Craniopharyngioma Registry revealed that preoperative HI was specifically associated with the development of severe long-term obesity [23]. Many published studies have concluded that left ventricular hypertrophy is one of the cardiac complications of obesity [27–32]. Cardiovascular morbidity in patients with CP was 22%, while cardiovascular risk factors were found in 57% of patients with CP [13]. Excess adipose tissue and augmented fat-free mass in obesity result in increased metabolic demand and lead to increases in blood volume and cardiac output [33]. This rise in blood volume leads to an increased venous return to both ventricular chambers, resulting in chamber enlargement, increasing wall tension, myocardial mass, and left ventricular hypertrophy [34].

Our study demonstrated that the degree of obesity in CP patients is correlated with an increased left ventricular wall thickness (IVSd and LVPWd). These results are consistent with several publications postulating that obesity is associated with interventricular wall and posterior wall thickness [31, 35, 36]. However, the observed positive correlation between BMI SDS and IVSd was stronger than the correlation between BMI SDS and LVPWd. Accordingly, we speculate that obesity affects the thickness of the interventricular septum more than that of the posterior wall, which supports previous studies observing the same phenomenon [37]. Furthermore, we observed a positive correlation between HL and increased IVSd and LVPWd. HLs leading to structural damage to medial and posterior hypothalamic nuclei result in hyperphagia and rapid weight gain [38]. We conclude that excessive weight gain in CP patients with HL leads to increased IVSd and LVPWd thickness. In our study, we found a trend towards a larger mean LVIDd in the group with obesity than in the non-obese group; but this trend did not reach statistical significance (51.7 mm vs. 43.49 mm, *p* = 0.057). Moreover, our study demonstrated that LVIDd positively correlated with the duration of disease, which supports previous research observing a positive correlation between the duration of morbid obesity and LVIDd [39]. The left and right ventricular functions of all CP patients in our study, as determined by EF, FS, and TAPSE, were within the normal range, which indicated that all patients had a normal systolic function of both ventricles. These findings were supported by previous studies [27, 30, 35, 36, 40, 41].

With regard to the endocrine aspects, we could demonstrate that cardiac remodeling was related to sex steroid replacement therapy, BMI SDS, and male gender. Previous studies demonstrated that sex steroids contributed to gender differences in cardiac remodeling. The majority of studies suggested that the female gender was associated with more favorable cardiac adaptations [42–44]. Moreover, in recent studies, increases in ventricular dimensions were reported more frequently in males compared to that in females [45]. Over 40% of the total testosterone in circulation is bound to the sex hormone–binding globulin (SHBG). The albumin-bound testosterone and unbound or free testosterone represent the major bioavailable male sex steroids [46]. In male mice, testosterone levels were found to be related to an increased left ventricular dimension [47], whereas increasing BMI was associated with decreased SHBG levels [48–50]. We speculate that lower SHBG levels in severely obese CP patients might result in an increased bioavailability of the circulating testosterone, which would result in increased LVIDd. Due to low levels of SHBG in obese and insulin-resistant patients [51], we suggest that patients with these conditions should be evaluated for bioavailable testosterone concentrations instead of total testosterone levels to prevent an over-supplementation with sex steroids.

IR is a condition in which the target cell shows a reduced response to insulin. IR is strongly associated with obesity [52, 53]. In our study, we could demonstrate that patients with decreased insulin sensitivity presented with increased IVS and LVPW thickness. Moreover, IVS thickness was related to IR and BMI. IR is known to be the determinant of left ventricular wall thickness [54–58]. The reduced insulin-like growth factor-1 (IGF-1) level in insulin-resistant patients has been reported to be associated with the higher circulating levels of the growth hormone and insulin resulting in cardiac hypertrophy [55]. Accordingly, we hypothesize that ventricular wall thickness is associated with the degree of obesity and IR. Left ventricular wall dilatation is correlated with the duration of disease, sex steroid replacement, higher BMI, and male gender.

Obesity leads to cardiac morbidity through several mechanisms. Increased BMI results in rising of cardiac output which causes cardiac remodeling and elevated blood pressure [59]. However, in our study, no direct correlation between BMI and hypertension was observed (*r* = − 0.092, *p* = 0.593).

Our study indicates that cardiac remodeling in CP patients is very complex and involves different aspects. Early identification of cardiac remodeling in patients with CP and obesity is important because initial prevention and treatment could modify the disease process resulting in improvement of the cardiac remodeling [31]. We recommend the implementation of TTE screening in all CP patients with obesity for early detection of cardiac remodeling, and systolic and diastolic dysfunction, especially in adult CP patients with severe obesity. In CP patients with technical limitations of TTE due to severe obesity, cardiac MRI could be considered a potentially more sensitive diagnostic technique. We suggest carrying out cardiac MRI (1) only in CP patients with TTE technical limitation due to obesity, (2) in CP patients not requiring general anesthesia for the diagnostic procedure of cardiac MRI, and (3) only in the setting of clinical trials. Larger studies and cost-benefit analyses are required to confirm this recommendation.

The limitations of our study include the low number of cases with severe obesity in our pediatric subgroup and the lack of data on other cardiac risk factors, such as smoking and family history of cardiac disease. Moreover, only two adult patients were observed to present with hyperlipidemia. A slight increase in IVSd was noted in the first patient, and slightly increased LVIDd and LVPWd were noted in the second patient with hyperlipidemia. Further research in larger pediatric populations is required to more accurately determine obesity-related cardiovascular dysfunction after CP. Furthermore, the limitations of our cross-sectional study require that future research should incorporate longitudinal follow-up of TTE parameters in patients with CP and obesity, in order to track the progression of cardiac remodeling and to evaluate the individual pathological manifestations of cardiovascular disease in these patients.

We conclude that cardiac remodeling in patients with childhood-onset CP was correlated with the degree of hypothalamic obesity, disease duration, sex hormone replacement therapy, male gender, and IR. Early identification of cardiac dysfunction is recommended. Due to restrictions of ultrasound condition caused by the severe obesity of patients with CP, we suggest that additional methods such as cardiac MRI should be considered in patients with poor ultrasound condition. However, further studies on the sensitivity and specificity of cardiac MRI are warranted after CP, which is part of our planned future study in context of the Craniopharyngioma Registry.

## Data Availability

Data are available on request from the senior author (HLM) and study committee of KRANIOPHARYNGEOM 2000/2007.

## Abbreviations

BMI: Body mass index
CP: Craniopharyngioma
FS: Fractional shortening
HI: Hypothalamic involvement
HL: Hypothalamic lesion
IVSd: Thickness of interventricular septum in diastole
LVIDd: Left ventricular internal diameter in diastole
LVPWd: Left ventricular posterior wall thickness in diastole
MRI: Magnetic resonance imaging
SDS: Standard deviation score
TAPSE: Tricuspid annular plane systolic excursion
TTE: Transthoracic echocardiography
WHO: World Health Organization
